# Neurosteroids in Glioma: A Novel Therapeutic Concept

**DOI:** 10.3390/life14080975

**Published:** 2024-08-02

**Authors:** Ava Hogan, Melike Mut

**Affiliations:** 1 Department of Neuroscience, University of Virginia, Charlottesville, VA 22903, USA; avf7mr@virginia.edu; 2 Department of Neurosurgery, University of Virginia, Charlottesville, VA 22903, USA

**Keywords:** neurosteroids, glioma, glioblastoma, surgical intervention, therapeutic targets, neuro-oncology

## Abstract

Glioma, a diverse group of brain and spinal cord tumors arising from glial cells, is characterized by varying degrees of malignancy, with some types exhibiting highly aggressive behavior, rapid proliferation, and invasive growth patterns, posing significant therapeutic challenges. This review delves into the complex interactions between glioma cells, neurotransmitters, and neurosteroids, emphasizing their potential as therapeutic targets. Key neurotransmitters, like glutamate and gamma-aminobutyric acid (GABA), play crucial roles in glioma growth, invasion, and treatment response. This review examines the involvement of neurosteroids in glioma biology and explores innovative therapeutic strategies targeting these systems. It encompasses the biosynthesis and mechanisms of neurosteroids, interactions between gliomas and neurotransmitters, the spatial distribution of neurosteroid synthesis in gliomas, the role of ion channels, hormonal influences, enzyme modulation, and the neuroimmune system in glioma progression. Additionally, it highlights the potential of neurosteroids to modulate these pathways for therapeutic benefit.

## 1. Introduction and Methodology

Gliomas represent the most frequent class of malignant primary tumor, and they arise from the glial cell family of the central nervous system (CNS). The World Health Organization (WHO) classifies these tumors into Grades I–II (low-grade gliomas) and Grades III–IV (high-grade gliomas, HGGs), according to their aggressiveness and genetic identities [1,2]. HGGs, while relatively rare, are associated with disproportionately high mortality and morbidity, regardless of treatment strategies [2]. Glioblastoma (GBM), the most lethal HGG subtype, is classified as a WHO Grade IV isocitrate dehydrogenase wild type (IDH-wt). GBM accounts for over half of diagnosed gliomas, with an incidence of 5/100,000 people/year in Europe and North America, and a median survival of 14.6 months post-diagnosis [1,2]. This review explores the complex interactions of glioma cells, neurotransmitters (NTs), neurosteroids (NSs), and neuroimmune pathways, emphasizing their potential as therapeutic targets against the aggressive behavior, rapid proliferation, and invasive growth patterns typical of these tumors. Following a summary of the findings, we discuss therapeutic hypotheses and how they could supplement current pharmacological strategies. As gliomas are the most genetically variable and deadly brain tumors, we delineate future research directions specific to glioma behavior that hold potential for improving clinical practices.

## 2. An Introduction to Neurosteroids

### 2.1. Biosynthesis of Neurosteroids

**Figure 1:**
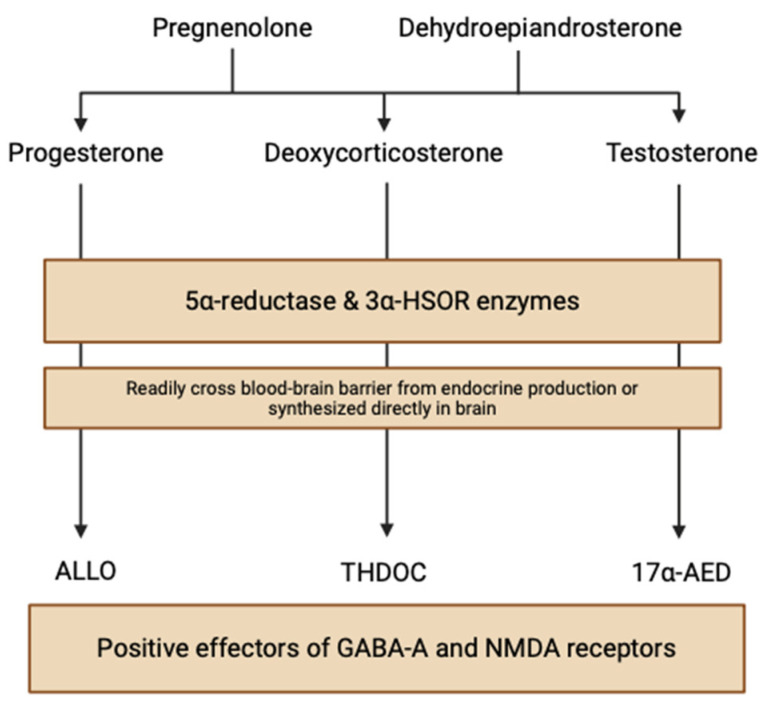
Neurosteroid biosynthetic pathways within the brain (created with BioRender.com (accessed on 20 June 2024)).

NSs are nonhydrolyzable lipid derivatives found in the brain, functioning to modulate neuronal synaptic interactions via non-genomic (electrical) and genomic (hormonal) pathways. This “neuroactive” steroid class is a broad and diverse group of hormones produced both in metabolic pathways and, significantly, locally within the human brain. Circulating steroid hormone precursors, like pregnenolone and dehydroepiandrosterone, are produced by cholesterol cleavage independent of endocrine gland supply, giving rise to progesterone, deoxycortisone, and testosterone steroid hormones (Figure 1) [3]. These steroids are reduced at 5α to NSs, like allopregnanolone (ALLO), allotetrahydrodeoxycorticosterone (THDOC), and androstanediol (17α-AED), by the enzymes 5α-reductase and 3α-hydroxysteroid oxidoreductase (3α-HSOR) in endocrine tissues [4]. NSs can quickly cross the blood–brain barrier due to their lipophilic and hydrophobic properties, accumulating in the brain from peripheral tissues. However, NS enzymes are also found in neuroectodermal tissues, myelin-synthesizing glial cells, and electrochemical-signaling neurons, indicating that neurosteroidogenesis can occur locally in the brain using peripherally synthesized precursors [3]. Stoffel-Wagner et al. (2003) found evidence of the local production of 5α-reductase and 3α-HSOR enzymes in the human hippocampus, neocortex, and amygdala [5]. Progesterone, a neurosteroid derived from the oxidation of pregnenolone by 3β-hydroxysteroid dehydrogenase, exhibits enzymatic mRNA expression in various brain regions including the cortex, hypothalamus, and cerebellum [6].

### 2.2. Neurosteroid Mechanism of Action

**Figure 2:**
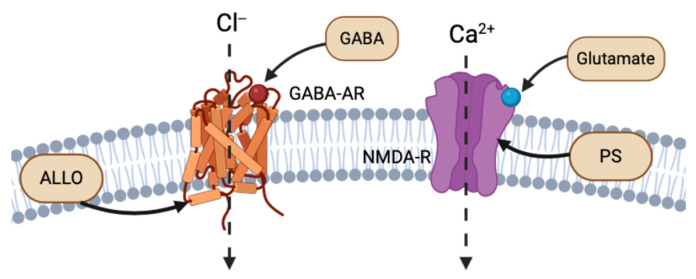
Example of intracellular lateral membrane diffusion of different NS binding to GABA-AR and NMDA-R (created with BioRender.com (accessed on 20 June 2024)).

The cortex, hypothalamus, and cerebellum associated with NS biosynthesis, NS enzymatic mRNA, and protein products are colocalized to glutamatergic excitatory neurons and GABAergic inhibitory neurons. Reddy (2004) discusses the importance of this discovery, as it implies that NSs are synthesized not only in the brain, but also specifically in the neurons that express their receptor targets [7]. Thus, NSs follow both autocrine and paracrine signaling mechanisms. NSs have the strongest affinity for and most rapid activity at electrical receptors and ion channels, altering the neuronal membrane potential. For instance, NSs, such as ALLO, THDOC, and pregnenolone sulfate (PS), act as allosteric modulators of the excitability of dendritic glutamatergic N-methyl-D-aspartate (NMDA) receptors and GABA-A receptors at the extracellular interface, or through autocrine intracellular lateral membrane diffusion (Figure 2) [7,8]. ALLO has also been shown to induce myelin formation and to promote neuron survival in excitotoxic environments [9].

Beyond the well-understood nongenomic effects of NSs, Rupprecht et al. (1993) explored how chronic effects of NS exposure may affect genomic steroid hormone receptors. NSs, such as progesterone, can either be synthesized intracellularly or enter by simple steroid diffusion through the plasma membrane. Specifically, the progesterone receptor located at the nuclear membranes in the hypothalamus and medial preoptic areas has been implicated for this NS mechanism of action [10]. 

### 2.3. Receptors at Plasma, Cytosolic, and Nuclear Membranes

The primary electrical receptors of NSs are glutamate and GABA type A receptors (GABA-AR), situated at the dendritic or postsynaptic membranes of neurons. GABA-ARs bind to the primary inhibitory NT GABA. Reddy (2010) reviewed the binding activation of an influx of chloride through chloride ion channels, ultimately decreasing membrane electrical potential and synaptic signaling (Figure 2) [4]. ALLO, THDOC, and 17α-AED NSs are positive effectors of GABA-AR, while sulfonated NS, such as PS and dehydroepiandrosterone sulfate (DHEAS), act as negative modulators of GABA-AR [4,11].

Glutamate is an excitatory NT that binds to excitatory NMDA receptors (NMDA-R), which increase the intracellular concentrations of calcium and increase membrane potential. The NS PS has shown to be a positive allosteric regulator of NMDA-R activity [12]. Most NSs bind to the extracellular binding site on their respective receptors, using the same mechanism as NT binding (Figure 2). However, similar to ALLO and THDOC intramembranous binding to GABA-R, PS has been shown to bind to the NMDA-R complex at a site separate from glutamate NTs and glycine modulators. Endogenous PS has been shown to bind to the transmembrane domain of NMDA-R, with both excitatory and inhibitory effects (Figure 2) [13].

**Figure 3:**
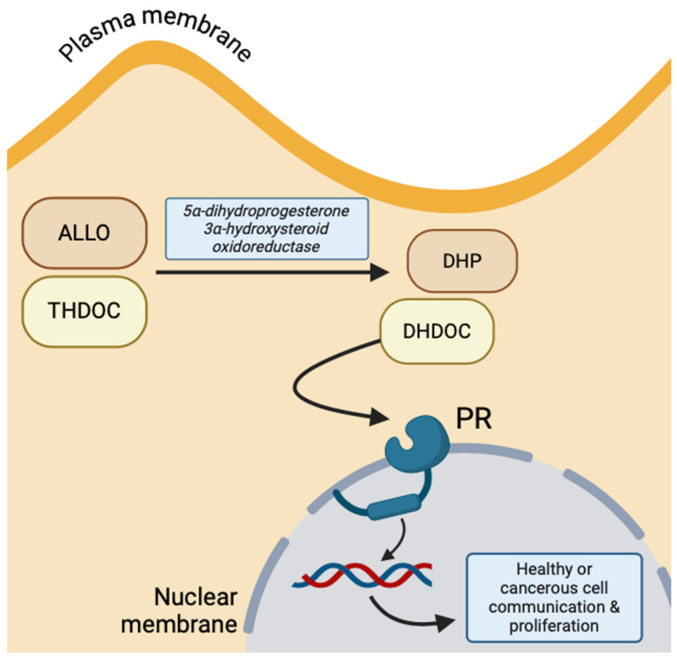
Intracellular progesterone receptor activation by oxidized ALLO and THDOC, implicated in healthy and malignant cell proliferation and communication (created with BioRender.com (accessed on 20 June 2024)).

Conversely, due to the lipophilic property of NS, they can cross the amphipathic neuronal membrane through simple diffusion and, thus, bind to targets on the cytosolic surface of nuclear membrane receptors. The primary example of NS hormonal receptors is the progesterone receptor (PR), which is activated by THDOC and ALLO intracellularly oxidized to 5α-dihydrodeoxycorticosterone (DHDOC) and 5α-dihydroprogesterone (DHP) by 5α-dihydroprogesterone 3α-hydroxysteroid oxidoreductase, respectively [10]. This receptor functions to regulate neuronal gene expression of growth factors and induce transcription of more PR (Figure 3) [14]. The predomination of genomic versus non-genomic NS effects depends on the number of progesterone receptors at the nuclear membrane, but both transmitter-gated ion channels and cytosolic gene expression are important for the communication between the plasma membrane and nucleus [10].

### 2.4. Neurotransmitters vs. Metabolic Hormones

Although both NTs and NSs act on GABA and glutamate systems, it is important to distinguish NSs from NTs. While NSs and NTs differ by their chemical constitution, they exhibit similar physiological effects. NTs are divided into three classes of amino acids, monoamines, or small neuropeptides, comprised of amino acids. There are twelve NTs identified, each with a well-defined chemical messenger role. NTs are released from vesicles and transmitted across synaptic clefts, functioning in a paracrine manner to bind and induce electrical changes in the postsynaptic neuron’s membrane potential or induce second-messenger cascades. In contrast, NSs are derivatives of steroid hormones which can act as NTs, specifically on GABA-AR and NMDA receptors [4]. Agís-Balboa et al. (2006) discussed the influence of NSs on the structure of the prefrontal cortex, as well as the enhancement of NSs on cerebellar, hippocampal, and cortical stem cell neurogenesis [8]. NSs differ from NTs as they are either synthesized intracellularly within the brain or diffuse through the blood–brain barrier from endocrine glands. Mechanistically, NSs operate through intracellular binding for genomic effects, rapid ligand-binding for ionotropic effects, and changes in membrane potential or composition [8]. 

## 3. Tumor Interactions

### 3.1. Gliomas and Neurotransmitters

Gliomas are the most common brain tumors, with genetic mutations that disrupt normal cell functions and lead to uncontrolled growth and infiltration of neural circuits. Gliomas arise from uncontrolled astrocyte, oligodendrocyte, or ependymal glial cell cycle divisions and growth. This abnormal proliferation, called tumorigenesis, pushes the glioma into healthy nervous system tissue and causes compression, as the CNS is confined by skeletal structures [15]. Glioma cells retain much of the highly communicative properties of healthy glial cells, allowing for interactivity and control of the peritumoral surrounding cell environment. This can extend into invasion of adjacent white matter tracts, or utilization of the lymphatic or venous system for transport to other parts of the body, or directly transverse the dura mater membrane to the skull. The spread of tumor outside of the original organ or tissue is called metastasis, a hallmark of stage IV glioma [2,15].

Recent research suggests a fascinating link between gliomas and NTs, the brain’s communication system. GBM, the most aggressive form and final stage of glioma, appears to exploit the brain’s natural plasticity. Studies show that gliomas can manipulate electrical activity, as well as the genetic and hormonal makeup of surrounding neurons [15]. Healthy neurons near the glioma, known as peritumoral neurons, can be tricked into supporting glioma growth by releasing factors, like neuroligin-3 (NLGN3) [16,17]. This highlights GBM’s ability to hijack the brain’s electrical communication for its own benefit, but the specific role of the nervous system in creating these altered regions remains under investigation. Understanding how gliomas interact with NTs, like glutamate and GABA, and by extension NS modulation, is crucial for developing new treatment strategies [18,19]. These key chemical messengers play complex roles in glioma biology, influencing tumor growth, invasion, and response to therapies.

### 3.2. Spatial Distribution of Neurosteroids and Gliomas

Mapping the physical location of NS synthesis and gliomas is essential. The human brain, a complex and intricate steroidogenic organ, contains 86 billion interconnected neurons segmented into distinct anatomical structures. Although gliomas can arise from anywhere in the CNS, they are most frequently found in the fronto-temporal cerebral hemispheres, areas that are also implicated in local neuro-synthesis of NSs [20]. This overlap indicates a possible relationship between the biosynthesis of NSs and primary locations of glioma development. Specific enzymes, like 5α-reductase, 3α-HSOR, 3β-hydroxysteroid dehydrogenase, and 3β-hydroxysteroid dehydrogenase/delta 5-delta 4 isomerase, which are catalysts for NS synthesis, have localized production in the same brain regions as gliomas [5,6]. Additionally, NSs are synthesized in various brain cells including oligodendrocytes, astrocytes, Schwann cells, Purkinje cells, hippocampal neurons, retinal amacrine, and ganglion cells, suggesting their involvement in multiple cancer types [4]. NSs can attenuate the migratory, invasive, and proliferative nature of gliomas.

### 3.3. Gliomas and Toll-Like Receptors

The neuroimmune system can be used to elevate the cytotoxic effects of gliomas beyond electrical stimulation. Pattern-recognition receptors, specifically Toll-like receptors (TLRs), function to recognize damage-associated molecular patterns intracellularly, and to recruit myeloid-differentiating factor response factors (MyD88) for the translation of nuclear (NF) and interferon (INF) transcription factors, resulting in subsequent activation of the innate cytokine proinflammatory defense and adaptive immune responses [21]. Essential to glioma’s abuse of TLR neuroimmune pathways is the ability to induce or reduce inflammation in the peritumoral microenvironment, as membrane TLRs are expressed both on circulating immune cells and glioma cells. Glioma cells can downregulate microenvironmental TLRs, which serves to both suppress the body’s innate immune system and protect glioma cells from adaptive immune system targeted destruction, while TLR overexpression in gliomas attenuates their proliferation and migration [22]. Within the family of TLRs, TLR-2, TLR-4, and TLR-9 stand out with unique expression patterns in glioma cell lines, while TLR-7 and 8 remain absent in gliomas [23,24]. Additionally, circulating immune cells (e.g., macrophages) and resident immune cells (e.g., microglia) with overexpressed TLR-2 and 5 were more successful in infiltrating and accumulating inflammatory immune responses against glioma invasion [25]. Furthermore, TLR-4 was found to be overexpressed in gliomas and correlated with increased cellular proliferation, while glioma U251 cell lines treated with the TLR-4 lipopolysaccharide (LPS) ligand had enhanced tumor growth and decreased survival [26].

## 4. Intersection of Neurosteroids and Gliomas

### 4.1. Glutamate, Neurosteroids, and Gliomas

Glutamate, the brain’s primary excitatory NT, plays a crucial role in glioma progression. Glioma cells release glutamate at excitotoxic concentrations, promoting tumor growth and invasion. Elevated extracellular glutamate levels activate receptors and transporters, increasing tumor cell proliferation and migration [27,28]. Glutamate transporters, particularly the excitatory amino acid transporter 2 (EAAT2), are essential for maintaining glutamate homeostasis. Glioma cells often downregulate EAAT2, resulting in higher extracellular glutamate levels [29]. Additionally, glutamate receptors, including ionotropic NMDA and AMPA receptors, are involved in signaling pathways that drive glioma tumorigenesis [30].

**Figure 4:**
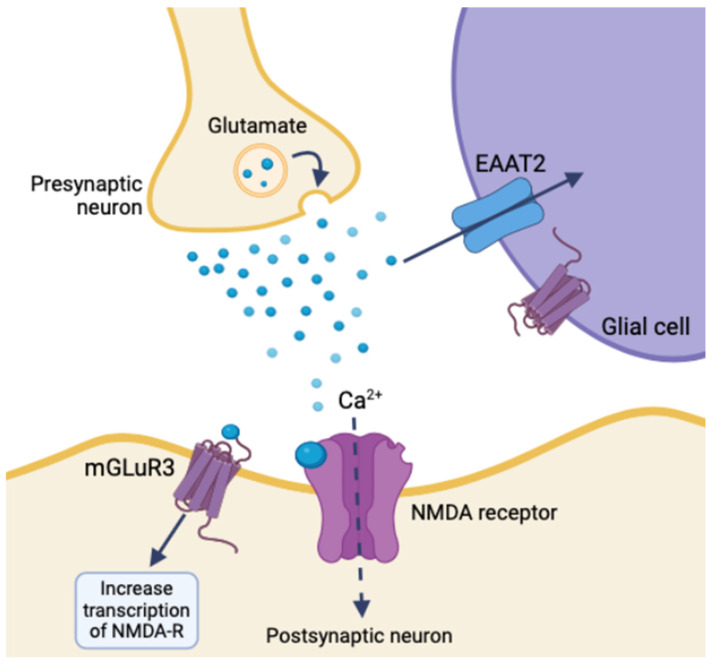
Ionotropic effects of glutamate release and glial synaptic clearing using EAAT2 in healthy tissue (created with BioRender.com (accessed on 20 June 2024)).

The GLUGLIO trial, a phase Ib/II randomized study, explores repurposing glutamate signaling inhibitors in combination with chemoradiotherapy for newly diagnosed GBM patients. This approach aims to reduce the tumor-promoting effects of glutamate by inhibiting its signaling pathways [31]. NSs could potentially enhance the efficacy of these inhibitors by further modulating glutamate receptor activity and reducing excitotoxicity. Negative allosteric modulators of metabotropic glutamate receptor 3 (mGluR3) show promise in targeting the stem-like phenotype of GBM, potentially reducing tumor aggressiveness [32]. mGluR3 increases synaptic transmission and overall neuronal excitability by coordinating with NMDA receptors and activating the transcription of other glutamate receptors. Glial glutamate receptors mediate synaptic plasticity using EAAT2, mGluR3, and ionotropic neuron receptors. Disrupting mGluR3 targets glioma stem-like cells and diminishes glutamatergic metabotropic crosstalk with the ionotropic receptor, illustrating the relationship between glutamate and excitatory enhancement in glioma cells [32]. NSs regulate the expression and function of NT transporters, like EAAT2 for glutamate uptake, altering extracellular NT levels and impacting tumor cell behavior (Figure 4) [29]. An inhibition of EAAT2 results in a buildup of extracellular glutamate, leading to neuron death. Glioma cells utilize a similar mechanism by under-expressing EAAT2 and increasing glutamate release, creating a toxic microenvironment, increasing astrocyte EAAT2-deficiency, and conferring survival and migratory advantages [29].

### 4.2. GABA, Neurosteroids, and Gliomas

GABA, the brain’s primary inhibitory NT, plays intricate roles in gliomas. Traditionally recognized for its inhibitory functions, GABA can exhibit paradoxical excitatory effects on GBM cells due to alterations in GABA receptor expression and signaling pathways [33]. These complex interactions significantly influence GBM biology and present potential therapeutic targets. GABA receptors, particularly GABA-B receptors, are critical in GBM cell proliferation and invasion. The upregulation of GABA-B receptors in GBM cells has been linked to increased tumor growth and resistance to apoptosis [33]. Furthermore, the p2 and θ subunits of the GABA-A receptor have increased expression in gliomas, supporting the notion that gliomas have distinct GABA channels related to patient survival [34]. NSs, like ALLO and tetrahydroprogesterone, are potent intracellular modulators of GABA-A receptors [8,35], enhancing GABAergic inhibitory effects and potentially reducing glioma cell proliferation and invasiveness, suggesting a promising avenue for therapy.

GABA typically exerts inhibitory effects on neuronal activity, but in gliomas, the expression and function of GABA receptors are altered. NSs can enhance GABA-A receptor activity, potentially restoring inhibitory signaling and reducing glioma cell proliferation and invasion [33,36]. This interaction suggests that NSs could be harnessed to counteract the aberrant GABAergic signaling observed in glioma. These findings highlight the therapeutic potential of modulating GABAergic signaling in glioma to improve patient outcomes.

### 4.3. Ion Channels, Neurosteroids, and Gliomas

Ion channels, including chloride (Cl^−^) and potassium (K^+^) channels, play significant roles in glioma cell migration and invasion. These channels help maintain cellular homeostasis and regulate cell volume, which is critical for tumor cell motility [37,38]. NSs influence the function of various ion channels, including Cl^−^ and K^+^ channels, involved in cell volume regulation and migration. By modulating these channels, NSs could affect the migration and invasiveness of glioma cells, providing another potential therapeutic target [39,40].

Calcium-permeable channels, like transient receptor potential canonical (TRPC1) channels and calcium-activated potassium channels, are critical in glioma cell signaling, proliferation, and migration [41,42]. The ability of NSs to modulate and sensitize these ion channels suggests they could alter glioma cell behavior through calcium signaling pathways [43]. This interaction highlights the potential for NSs to impact cell proliferation and migration, offering promising avenues for glioma treatment strategies. Blocking ion channels has been shown to reduce glioma cell invasion. For instance, Cl^−^ and K^+^ ion channel blockers can inhibit glioma cell migration and invasion, suggesting potential therapeutic benefits [40]. Additionally, calcium-permeable channels, like TRPC1, are implicated in glioma cell signaling and could be targeted to disrupt tumor growth [41]. Calcium influx through TRPC1 activates voltage-gated chloride channels, initiating volume changes and highlighting the ion-powered mechanism of tumor invasion and chemotaxis [41].

### 4.4. Toll-Like Receptors, Neurosteroids, and Gliomas

**Figure 5:**
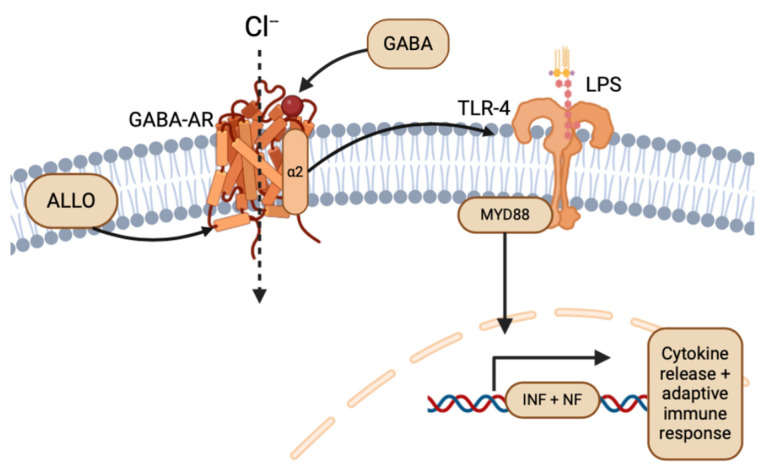
Overview of ALLO and GABA-AR in the TLR-4 pathway (created with BioRender.com (accessed on 20 June 2024)).

While glioma’s hijacking and evasion of the neuroimmunology system through TLRs has promise for direct therapy through immunopharmacology [22,44], an indirect approach targeting the ligands or agonists of TLR pathways also serves as a potential therapeutic avenue. Particularly, the NS ALLO has been shown to inhibit inflammatory signals induced by TLR-4 [45], indicating the potential for ALLO to reduce the effects of glioma on the peritumoral environment, if targeted. As mentioned previously, ALLO potentiates the inhibitory activity of GABA-AR, having sedative and anti-convulsant effects on the peritumoral environment and CNS [8,35]. In neuron and glial cells, TLR-4 signaling is associated with GABA-AR α2 subunit activation upstream, and subsequent TLR4 pathway-induced inflammation (Figure 5) [45,46]. Following the dual expression of TLR in local immune cells and glioma, ALLO and other NSs could play complex roles in both preventing glioma immune system evasion, inhibition of TLRs of peritumoral cells, and detrimental abuse of inflammation through agonistic and antagonistic effects [22,47]. Because the innate immune system and TLRs are so essential for antitumor responses, the ability of gliomas to express TLRs remains an important target for treatment. 

### 4.5. Hormonal Influences, Neurosteroids, and Gliomas 

The enzyme for the rate-limiting step of steroidogenesis is transcriptionally active in glioma cells but not in healthy glial cells, implicating early precursors of neurosteroidogenesis in glioma’s altered expression and concentration of NSs [48]. Other NS precursors, like progesterone and testosterone, have been shown to increase pre-glial stem cell and GBM proliferation [49]. Stem cells derived from the cerebral cortex and pre-glial cells exhibit decreased regulation of mitotic cell-cycle genes and increased proliferation in the presence of increasing concentrations of ALLO [9]. In high-plasticity glioma stem cells, the genetic expression of the progesterone receptor (PR) was higher than in healthy stem cells, implicating progesterone in glioma stem cell proliferation [49]. PR is expressed at higher levels in both reproductive and non-reproductive cancers, like GBM [50]. Bello-Alvarez et al. (2022) investigated the extranuclear mechanisms activated by PR overexpression, focusing on molecular interactions between GBM cells and PR-neurosteroid ligands. PR expression enhances cancer hallmarks, such as proliferation, migration, and invasion of breast cancer cells, but the specific molecular changes caused by PR expression in GBM cells remain unclear [50].

**Figure 6:**
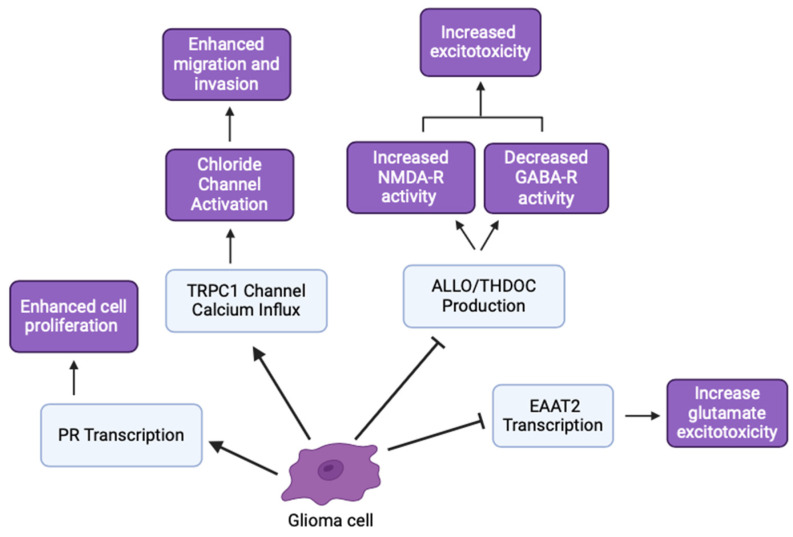
Various glioma inhibitory and excitatory effects involved in cancer hallmarks (created with BioRender.com (accessed on 20 June 2024)).

Hormonal differences significantly influence GBM incidence and progression. Higher levels of testosterone-derived estradiol in GBM cells promote invasive and migratory morphologies, indicating a role for sex hormones in glioma behavior [51]. Enzymes, like 5α-reductase and 3α-HSOR, which are involved in neurosteroid synthesis, are upregulated in GBM cells. Inhibition of these enzymes reduces the synthesis of androgenic metabolites, suggesting that targeting these enzymes could inhibit GBM growth [52,53]. The effects of NS and tumor proliferation on hyperexcitability and decreased inhibitory signaling have important implications in seizures and epileptic activity [54]. Stoffel-Wagner et al. (2001) found that ALLO levels and 5α-reductase/3α-HSOR mRNA expression differed between hippocampal and cortical tissue in patients with gliomas [5,18]. Additionally, antiepileptic drug regimens that modulate NSs concentrations in epileptic patients had similar outcomes for glioma patients [54]. By modulating hormone levels and NSs synthesis, we can explore therapeutic strategies that impact glioma cell behavior and potentially improve patient outcomes (Figure 6).

## 5. Discussion 

The intricate relationship between NSs and glioma progression presents a promising frontier for therapeutic advancements. NSs, by modulating key NT systems, notably glutamate and GABA, influence glioma growth dynamics and the associated neural disruptions. Here, we explore their dual roles and potential as targets in innovative treatment strategies.

NSs serve as potential glutamate antagonists, counteracting excitotoxicity—a significant factor in tumor proliferation. By diminishing glutamate’s harmful effects, NSs offer a therapeutic pathway to curb tumor growth, leveraging their role to protect neuronal integrity while inhibiting glioma cells [55].

Gender disparities in glioma incidence suggest that hormonal variations play a crucial role. NSs, through their interaction with hormone receptors, may influence these disparities, offering insights into gender-specific therapeutic approaches. The modulation of steroid hormone levels and receptor expressions by NSs can impact tumor behavior differently across genders, suggesting a tailored approach in therapeutic interventions [4].

NSs also interact with GABA-A receptors, affecting glioma development by altering neuronal excitability. This interaction is linked to symptoms, such as seizures, common in glioma patients, pointing to NSs’ potential to mitigate both neurological symptoms and tumor growth through modulating GABAergic signaling [34].

The dynamic interplay between the nervous system and tumor growth is elaborated, where neural activity is shown to promote tumor growth through the secretion of growth factors, like NLGN3. This underscores the significance of NSs in modulating interactions between neuronal and glial cells, making them pivotal in the neural–glioma interface [17]. Venkatesh et al. (2015) research highlights how glioma cells exploit neural circuits for growth, emphasizing NSs’ role in modulating the tumor microenvironment [16]. By influencing NT systems, such as glutamate and GABA, NSs affect glioma biology, suggesting that targeting these pathways could lead to effective therapeutic strategies. 

Allopregnanolone exemplifies the dual roles of NSs, where different concentrations can either promote or inhibit tumor progression. This nuanced behavior indicates the need for precise modulation of NS levels in therapy. Furthermore, enzymes involved in neurosteroid synthesis, like 5α-reductase and 3α-HSOR, which are expressed in glioma cells, contribute to tumor growth by modulating androgenic metabolites. NSs modulate NT systems, impacting glioma growth and neural function. They can act as both agonists and antagonists on GABA-A receptors and influence glutamatergic signaling. For instance, allopregnanolone can modulate GABAergic activity, which is significant in managing glioma-associated symptoms, like seizures [48,56,57]. Incorporating findings from Patel et al. (2015), who discuss neuron–glia interactions in epilepsy, enhances the understanding of glioma-induced neurological disruptions. This research underscores potential NS targets within epileptogenic processes, further emphasizing NSs’ therapeutic potential in managing both tumor growth and epilepsy [58]. 

As discussed previously, the electrical and inflammatory nature of gliomas on TLRs through enhanced neurosteroid production functions to induce epileptic activity and inflammation in healthy brain tissue. While these are understood consequences of GBM activity, TLR proinflammatory immune pathways emerge as a strong potential therapeutic target. Furthermore, TLR-4 at the cell membrane binds to its bacterial LPS ligand and recruits myeloid-differentiating factors, like MyD88, for an infection-induced inflammatory cascade (Figure 5). TLR-4 signaling is unique in neuron cells, as it involves GABA-AR subunits upstream of TLR4 activation [45,46]. While the implication and interaction in glioma biology remains unelucidated, the dual inhibitory effect of ALLO and THDOC through GABA-AR on TLR-4 proinflammation pathways remains an exciting venture for future research into molecular targets of glioma and GBM hallmarks [45,59]. Additionally, TLR-4 overexpression in precursor GBM cell lines was shown to reduce proliferation and differentiation, while under expression of TLR-4 in GBM lines was associated with increased apoptosis [23,60]. As such, TLR agonists represent potential immune modulators that could supplement other antitumoral treatments, while GBM-TLR antagonists could reduce glioma-induced inflammation [22]. 

Dexamethasone, a corticosteroid commonly used to reduce vasogenic edema in glioma patients, exerts its effects by stabilizing the blood–brain barrier and reducing inflammation. However, its immunosuppressive properties and impact on overall survival in glioma patients raise concerns [22]. Interestingly, NSs also play a role in modulating inflammation and neuronal excitability, such as dexamethasone. Both dexamethasone and other neurosteroids, like ALLO, influence GABAergic signaling, although their mechanisms and outcomes can differ. While dexamethasone reduces edema and inflammation through genomic and non-genomic pathways, NSs can modulate NT systems to protect neurons and potentially inhibit glioma growth [61]. Additionally, neurosteroids may offer a more targeted approach with fewer systemic side effects compared to dexamethasone. By modulating specific NT systems and hormone receptors, neurosteroids could provide a therapeutic advantage in managing glioma progression and associated neurological symptoms [62]. 

NSs hold potential as regenerative agents due to their neuroprotective properties and ability to modulate NT pathways. Research suggests that allopregnanolone and similar compounds can promote neural regeneration and protect against excitotoxicity, which is beneficial in glioma treatment. Targeting neurosteroid pathways may offer a complementary approach to existing therapies, potentially improving patient outcomes [57,63].

### Future Research Directions

Investigating the specific roles of various NSs in gliomas is crucial for a deeper understanding of their individual effects on tumor biology, enabling researchers to pinpoint the most promising candidates for therapeutic targeting. Furthermore, combining NS modulators with established treatment modalities, such as surgery, radiation, and chemotherapy, could offer synergistic benefits, enhancing therapeutic efficacy. These combination therapy approaches are currently being explored in clinical trials, promising to revolutionize glioma, particularly GBM, treatment strategies. Moreover, while dexamethasone remains a crucial tool in managing glioma-associated edema, exploring the potential of NSs, like in TLR immune pathways, offers a complementary approach that could mitigate some of the adverse effects associated with long-term corticosteroid use. By leveraging the distinct yet overlapping pathways modulated by both dexamethasone and NSs, there is potential to develop more effective and nuanced therapeutic strategies for glioma patients. 

## 6. Conclusions

The exploration of NSs in gliomas offers promising avenues for innovative therapeutic strategies, leveraging the complex interplay between NS and NT systems and tumor biology. As ongoing research sheds light on the nuanced roles of NSs in modulating neural and tumor environments, there exists a substantial opportunity to translate these insights into effective clinical applications. By deepening our understanding of these intricate biological mechanisms, we can develop targeted therapeutic strategies that enhance treatment efficacy, curb glioma progression, and improve patient outcomes. Innovations in this field could lead to breakthrough therapies that not only suppress tumor growth and surrounding inflammation, but also mitigate the neurological impacts of gliomas, enhancing both the quality and effectiveness of patient care.
